# Potential therapeutic target identification in the novel 2019 coronavirus: insight from homology modeling and blind docking study

**DOI:** 10.1186/s43042-020-00081-5

**Published:** 2020-10-02

**Authors:** Olanrewaju Ayodeji Durojaye, Talifhani Mushiana, Henrietta Onyinye Uzoeto, Samuel Cosmas, Victor Malachy Udowo, Abayomi Gaius Osotuyi, Glory Omini Ibiang, Miapeh Kous Gonlepa

**Affiliations:** 1 School of Life Sciences, Department of Molecular and Cell Biology, University of Science and Technology of China, Hefei, China; 2 Department of Biochemistry, University of Nigeria, Nsukka, Enugu State Nigeria; 3 Department of Chemical Sciences, Coal City University, Emene, Enugu State Nigeria; 4 School of Chemistry and Material Sciences, Department of Chemistry, University of Science and Technology of China, Hefei, China; 5 Department of Biological Sciences, Coal City University, Emene, Enugu State Nigeria; 6 Institute of Metal Research, Chinese Academy of Sciences, Shenyang, China; 7 School of Earth and Space Sciences, University of Science and Technology of China, Hefei, China; 8 School of Public Affairs, Department of Public Administration, University of Science and Technology of China, Hefei, China

**Keywords:** Coronavirus, Proteinase, Replication, Ligand, Inhibitors

## Abstract

**Background:**

The 2019-nCoV which is regarded as a novel coronavirus is a positive-sense single-stranded RNA virus. It is infectious to humans and is the cause of the ongoing coronavirus outbreak which has elicited an emergency in public health and a call for immediate international concern has been linked to it. The coronavirus main proteinase which is also known as the 3C-like protease (3CLpro) is a very important protein in all coronaviruses for the role it plays in the replication of the virus and the proteolytic processing of the viral polyproteins. The resultant cytotoxic effect which is a product of consistent viral replication and proteolytic processing of polyproteins can be greatly reduced through the inhibition of the viral main proteinase activities. This makes the 3C-like protease of the coronavirus a potential and promising target for therapeutic agents against the viral infection.

**Results:**

This study describes the detailed computational process by which the 2019-nCoV main proteinase coding sequence was mapped out from the viral full genome, translated and the resultant amino acid sequence used in modeling the protein 3D structure. Comparative physiochemical studies were carried out on the resultant target protein and its template while selected HIV protease inhibitors were docked against the protein binding sites which contained no co-crystallized ligand.

**Conclusion:**

In line with results from this study which has shown great consistency with other scientific findings on coronaviruses, we recommend the administration of the selected HIV protease inhibitors as first-line therapeutic agents for the treatment of the current coronavirus epidemic.

## Background

The first outburst of pneumonia cases with unknown origin was identified in the early days of December 2019, in the city of Wuhan, Hubei Province, China [1]. Revelation about a novel beta coronavirus currently regarded as the 2019 novel coronavirus [2] came up after a high-throughput sequencing of the viral genome which exhibits a close resemblance with the severe acute respiratory syndrome (SARS-CoV) [3]. The 2019-nCoV is the seventh member of enveloped RNA coronavirus family (subgenus sarbecovirus, Orthocoronavirinae) [3], and there are accumulating facts from family settings and hospitals confirming that the virus is most likely transmitted from person-to-person [4]. The 2019-nCoV has also recently been declared by the World Health Organization as a public health emergency of international concern [5] and as of the 5th of February 2020, over 24,000 cases has been confirmed and documented from laboratories around the world [6] while more than 28,000 of such cases were documented in China through laboratory confirmation as of the 6th of February 2020 [7]. Despite the fast rate of global spread of the virus, the characteristics clinically peculiar to the 2019-nCoV acute respiratory disease (ARD) remain unclear to a very large extent [8].

Over 8000 infections and 900 deaths were recorded worldwide in the summer of 2003 before a successful containment of the severe acute respiratory syndrome wave was achieved as the disease itself was also a major public health concern worldwide [9, 10]. The infection that led to a huge number of death cases was linked to a new coronavirus also known as the SARS coronavirus (SARS-CoV). Coronaviruses are positive-stranded RNA viruses and they possess the largest known viral RNA genomes. The first major step in containing the SARS-CoV-lined infection was to successfully sequence the viral genome, the organization of which was found to exhibit similarity with the genome of other coronaviruses [11].

The main proteinase crystal structure from both the transmissible gastroenteritis virus and the human coronavirus (hCoV 229E) has been determined with the discovery that the enzyme crystal structure exists as a dimer and the orientation of individual protomers making up the dimer has been observed to be perpendicular to each other. Each of the protomers is made up of three catalytic domains [12]. The first and second domains of the protomers have a two-β-barrel fold that can be likened to one of the folds in the chymotrypsin-like serine proteinases. Domain III have five α-helices which are linked to the second domain by a long loop. Individual protomers have their own specific region for the binding of substrates, and this region is positioned in the left cleft between the first and second domain. Dimerization of the protein is thought to be a function of the third domain [13]. The main proteinase of the SARS CoV is known to be a cysteine proteinase which has in its active site, a Cysteine-Histidine catalytic dyad. Conservation of the SARS CoV main proteinase across the genome sequence of all SARS coronaviruses is very high, likewise the homology of the protein to the main proteinase of other coronaviruses. On the basis that high similarity exists between the different coronavirus main proteinase crystal structures and the conservation of almost all the amino acid residue side chains involved in the dimeric state formation, it was proposed that the only biologically functional form coronavirus main proteinase might be is its existence as a dimer [14]. More recently, Chen et al. in his study which involved the application of molecular dynamic simulations and enzyme activity measurements from a hybrid enzyme showed that the only active form of the proteinase is in its dimeric state [15].

Recent studies based on the sequence homology of the coronavirus main proteinase structural model with TGEV as well as the solved crystal structure has involved the docking of substrate analogs for the virtual screening of natural products and a collection of synthetic compounds, alongside approved antiviral therapeutic agents in the evaluation of the coronavirus main proteinase inhibition [16]. Some compounds from this study were identified for the inhibitory role played against the viral proteinase. These compounds include the L-700,417, which is an HIV-1 protease inhibitor, calanolide A, and nevirapine, both of which are reverse transcriptase inhibitors, an inhibitor of the α-glucosidase named glycovir, sabadinine, which is a natural product and ribavirin, a general antiviral agent [17]. Ribavirin was shown to exhibit an antiviral activity in vitro, at cytotoxic concentrations against the SARS coronavirus. At the start of the first outbreak of the SARS epidemic, ribavirin was administered as a first-line of defense. The administration was as a monotherapy and in combination with corticosteroids or the HIV protease inhibitor, kaletra [18]. According to reports from a very recent research conducted by Cao et al., where a total of a 199 laboratory-confirmed SARS-CoV-infected patients were made to undergo a controlled, randomized, open-labeled trial in which 100 patients were assigned to the standard care group and 9 patients assigned to the lopinavir–ritonavir group. 48.4% of the patients in the lopinavir–ritonavir group (46 patients) and 49.5% of the patients in the standard care group (49 patients) exhibited serious adverse events between randomization and the 28th day. The exhibited adverse events include acute respiratory distress syndrome (ARDS), acute kidney injury, severe anemia, acute gastritis, hemorrhage of lower digestive tract, pneumothorax, unconsciousness, sepsis, acute heart failure etc. Patients in the lopinavir–ritonavir group in addition, specifically exhibited gastrointestinal adverse events which include diarrhea, vomiting, and nausea [19].

**Fig. 1:**
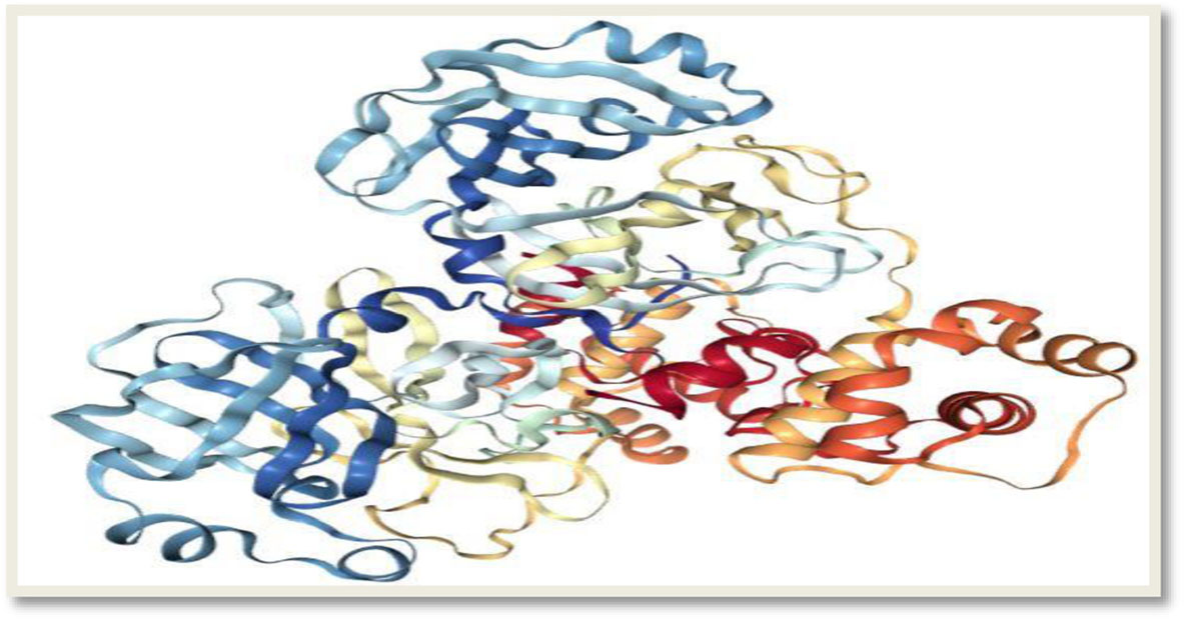
Dimeric crystal structure of the SARS coronavirus main proteinase (source https://www.rcsb.org/3d-view/1UJ1/1)

Our current study took advantage of the availability of the SARS CoV main proteinase amino acid sequence to map out the nucleotide coding region for the same protein in the 2019-nCoV. Two selected HIV protease inhibitors (lopinavir and ritonavir) were then targeted at the catalytic site of the protein 3D structure which was modeled using already available templates. The predicted activity of the drug candidates was validated by targeting them against a recently crystalized 3D structure of the enzyme, which has been made available for download in the protein data bank. Lopinavir is an antiretroviral protease inhibitor used in combination with ritonavir in the therapy and prevention of human immunodeficiency virus (HIV) infection and the acquired immunodeficiency syndrome (AIDS). It plays a role as an antiviral drug and a HIV protease inhibitor. It is a member of amphetamines and a dicarboxylic acid diamide (Fig. 1).

## Methods

### Sequence analysis

The complete genome of the isolated Wuhan seafood market pneumonia virus (2019-nCoV) was downloaded from the GenBank database with an assigned accession number of MN908947.3. The nucleotide sequence of the full genome was copied out in FASTA format. The GenBank sequence database is an annotated collection of all nucleotide sequences which are publicly available with their translated protein segments and also open access. This database is designed and managed by the National Center for Biotechnology Information (NCBI) in accordance with the International Nucleotide Sequence Database Collaboration (INSDC) [20]. Nucleotides between the 10055 and 10972 sequence of the 2019-nCoV genome was selected as the sequence of interest. Translation of the nucleotide sequence of interest in the 2019-nCoV and the back-translation of the SARS CoV main proteinase amino acid sequence was achieved with the use of EMBOSS transeq and backtranseq tools, respectively [21]. Transeq reads one or more nucleotide sequences and writes the resulting translated sequence of protein to file while backtranseq makes use of a codon usage table which gives the usage frequency of individual codon for every amino acid [22]. For every amino acid sequence input, the corresponding most frequently occurring codon is used in the nucleotide sequence that forms the output. The corresponding amino acid sequence generated as a product of the transeq translation of the nucleotide sequence of interest had no stop codons and as such was used directly for protein homology modeling without the need for any deletion.

### Sequence alignment

Two sets of sequence alignments were carried out in this study. The first was the alignment between the translated nucleotide sequence copy of the 2019-nCoV genome which was used for the reference protein homology modeling and the amino acid sequence of the SARS CoV main proteinase while the second alignment was between the back-translated SARS CoV main proteinase nucleotide sequence and the 2019-nCoV full genome. The latter was used in mapping out the protein coding sequence in the 2019-nCoV full genome. These alignments were carried out using the Clustal Omega software package. Clustal Omega can read inputs of nucleotide and amino acid sequences in formats such as a2m/Fasta, Clustal, msf, phylip, selex, Stockholm, and Vienna [23].

### Model building

Template search with BLAST and HHBlits was performed against the SWISS-MODEL template library. The target sequence was searched with BLAST against the primary amino acid sequence contained in the SMTL. A total of 120 templates were found. An initial HHblits profile was built using the procedure outlined in Remmert et al. [24] followed by 1 iteration of HHblits against NR20. The obtained profile was then searched against all profiles of the SMTL. A total of 192 templates were found. Models were built based on the target-template alignment using ProMod3. Coordinates which are conserved between the target and the template are copied from the template to the model. Insertions and deletions are remodeled using a fragment library. Side chains were then rebuilt and finally, the geometry of the resulting model, regularized by using a force field [25].

### Selection of a reliable model

For the estimation of the protein structure model quality, we used the QMEAN (Qualitative Model Energy Analysis), a composite scoring function that describes the main aspects of protein structural geometrics, which can also derive on the basis of a single model, both global (i.e., for the entire structure) and local (i.e., per residue) absolute quality estimates [26]. An appreciable number of alternative models have been produced which formed the basis on which scores produced by the final model was selected. The QMEAN score was thus used in the selection of the most reliable model against which the consensus structural scores were calculated.

### Model quality estimation

MolProbity (version 4.4) was used as the structure-validation tool that produced the broad-spectrum evaluation of the quality of the target protein at both the global and local levels. It greatly relies on the provided sensitivity and power by optimizing the placement of hydrogen and all-atom contact analysis with complementary versions of updated covalent-geometry and torsion-angle criteria [27]. The torsion angles between individual residues of the target protein were calculated using the Ramachandran plot. This is a plot of the torsional angles [phi (φ) and psi (ψ)] of the amino acid residues making up a peptide. In the order of sequence, the torsion angle of N(i-1), C(i), Ca(i), N(i) is φ while the torsion angle of C(i), Ca(i), N(i), C(i+1) is ψ. The values of φ were plotted on the *x*-axis while the values of ψ were plotted on the *y*-axis [28]. Plotting the torsional angles in this way graphically shows the possible combination of angles that are allowed.

### Oligomeric state conservation

The quaternary structure annotation of the template is employed to model the target sequence in its oligomeric state. The methodology as proposed by Bertoni et al. [29] was supported on a supervised machine learning algorithm rule, support vector machines (SVM), which mixes conservation of interface, clustering of structures with other features of the template to produce a quaternary structure quality estimate (QSQE). The QSQE score is a number that ranges between 0 and 1, and it is a reflection of the accuracy expected of the inter-chain contacts for a model engineered based on a given template and its alignment. The higher score is an indication of a more reliable result. This enhances the GMQE score that calculates the accuracy of the 3D structure of the resulting model.

### 3D structure comparison

The 3D structural homology modeling of the 2019-nCoV genome translated segment was followed by a structural comparison with the SARS CoV main proteinase 3D structure (PDB: 1UJ1). This was achieved using the UCSF Chimera which is a highly extensible tool for interactive analysis and visualization of molecular structures and other like data, including docking results, supramolecular assemblies, density maps, sequence alignments, trajectories, and conformational ensembles [30]. High-quality animation videos were also generated.

### Secondary structure visualization

The amino acid constituents of the target protein secondary structures were colored and visualized in 3D using the Pymol molecular visualzer which uses OpenGL Extension Wrangler Library (GLEW) and FreeGLUT. The *Py* aspect of the PyMol is a reference to the programming language that backs up the software algorithm which was written in Python [31]. The percentage composition of each component making up the secondary structure was calculated using the Chou and Fasman Secondary Structure Prediction (CFSSP) server. This is a secondary structure predictor that predicts regions of secondary structure from an amino acid input sequence such as the regions making up the alpha helix, beta sheet, and turns. The secondary structure prediction output is displayed in a linear sequential graphical view according to the occurrence probability of the secondary structure component. The CFSSP implemented methodology is the Chou-Fasman algorithm, which is based on the relative frequency analyses of alpha helices, beta sheets, and loops of each amino acid residue on the basis of known structures of proteins solved with X-ray crystallography [32].

### Protein physiochemical parameters calculation

The ExPASy server calculates protein physiochemical parameters as a part of its sub-function, basically for the identification of proteins [33]. We engaged the function of the Protparam tool in calculating various physiochemical parameters in the model and template protein for comparison purposes. The calculated parameters include the molecular weight, theoretical isoelectric point, amino acid composition, extinction coefficient, instability index, etc.

### Molecular phylogenetic analysis by maximum likelihood method

The inference on evolutionary relationship was made utilizing the maximum likelihood methodology which is the basis of the JTT matrix-based model [34]. The corresponding consensus tree on bootstrap was inferred from a thousand replicates, and this was used to represent the historical evolution of the analyzed taxa. The tree branches forming partitions that were reproduced in bootstrap replicates of less than 50% were automatically collapsed. Next to every branch in the tree is the displayed percentage of tree replicates of clustered associated taxa in the bootstrap test of a thousand replicates. Initial trees were derived automatically for the search through the application of the Neighbor-Join and BioNJ algorithms to a matrix of pairwise distances calculated using a JTT model and followed by the selection of the most superior log likelihood value topology. The phylogenetic analysis was carried out on 12 amino acid sequences with close identity. The complete dataset contained a total of 306 positions. The whole analysis was conducted using the Molecular Evolutionary and Genetics Analysis (MEGA) software (version 7) [35].

### Ligand preparation and molecular docking protocol

2D structures of the experimental ligands were viewed from the PubChem repository and sketched using the ChemAxon software [36]. The sketched structures were downloaded and saved as mrv files which were converted into SMILES strings with the OpenBabel. The compounds prepared as ligands were docked against each of the prepared protein receptors using AutoDock Vina [37]. Blind docking analysis was performed at extra precision mode with minimized ligand structures. After a successful docking, a file consisting of all the poses generated by the AutoDock Vina along with their binding affinities and RMSD scores was generated. In the Vina output log file, the first pose was considered as the best because it has stronger binding affinity than the other poses and without any RMSD value. The polar interactions and binding orientation at the active site of the proteins were viewed on PyMol and the docking scores for each ligand screened against each receptor protein were recorded. The same docking protocol was performed against the SARS-CoV main proteinase 3D structure that was downloaded from the protein data bank with a PDB identity of 6m2n. Obtained outputs were visualized, compared, and documented for validation purpose.

## Results

### Sequence analysis

**Fig. 2:**
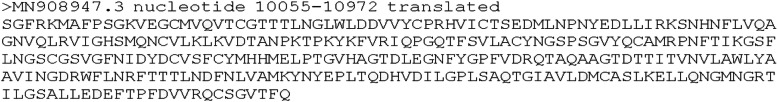
Translated region of the 2019-nCoV nucleotide sequence with the full sequence forming the target protein coding sequence

The full genome of the 2019-nCoV (https://www.ncbi.nlm.nih.gov/nuccore/MN908947.3?report=fasta) consists of 29903 nucleotides, but for the purpose of this study, nucleotides between 10055 and 10972 were considered to locate the protein of interest. The direct translation of this segment of nucleotides produced a sequence of 306 amino acids (Fig. 2). This amino acid count was reached after the direct translation of the nucleotide sequence of interest as there were no single existing stop codons hence, deletion of any form was needless.

### Sequence alignment

**Fig. 3:**
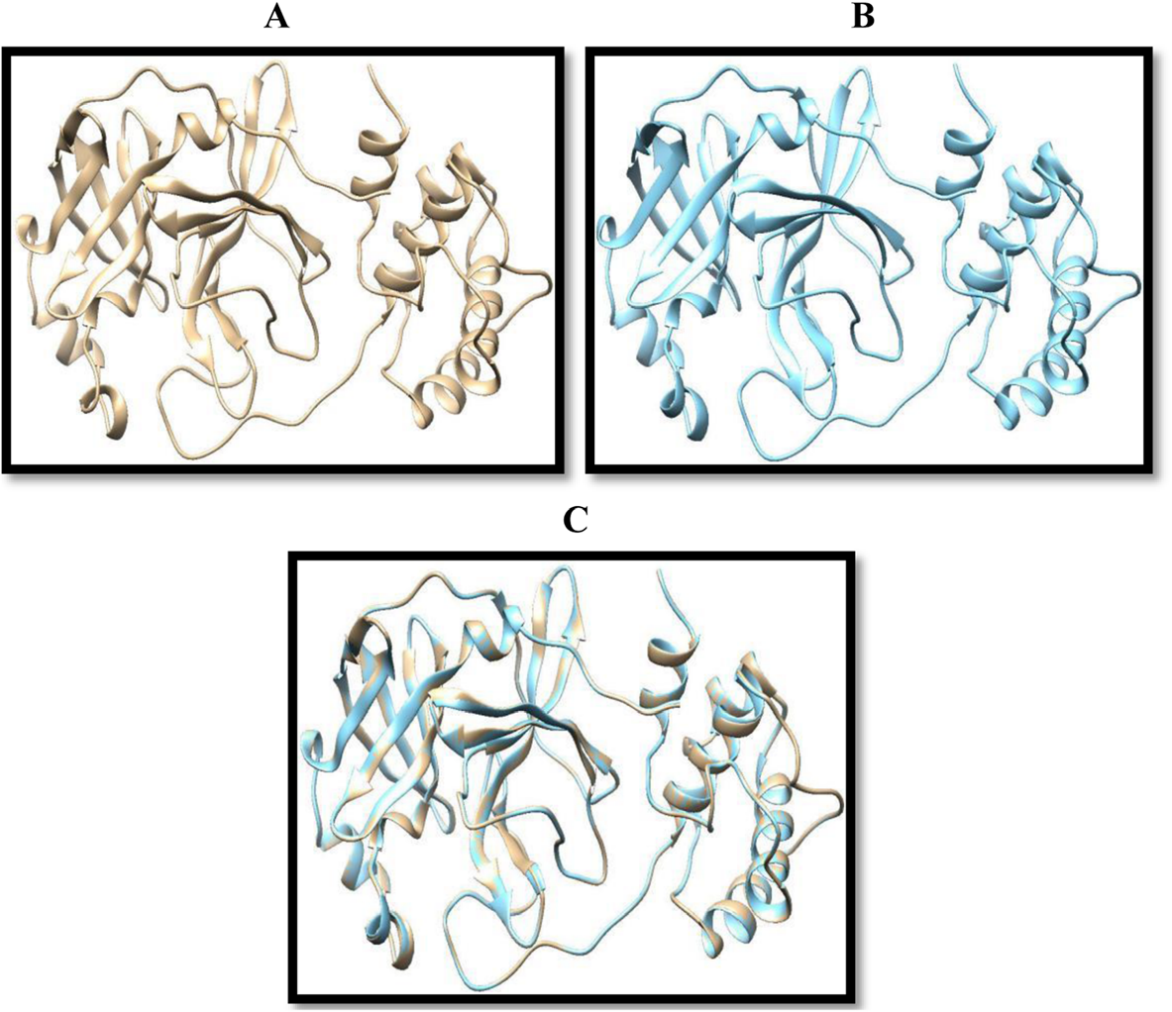
3D structures of the target and template protein, with the structural comparison. The target protein is represented at the top left region in grey color (**a**), while the template is on the top right in light blue color (**b**). The matching together of the two was depicted in the mixed color picture beneath (**c**)

**Fig. 4:**
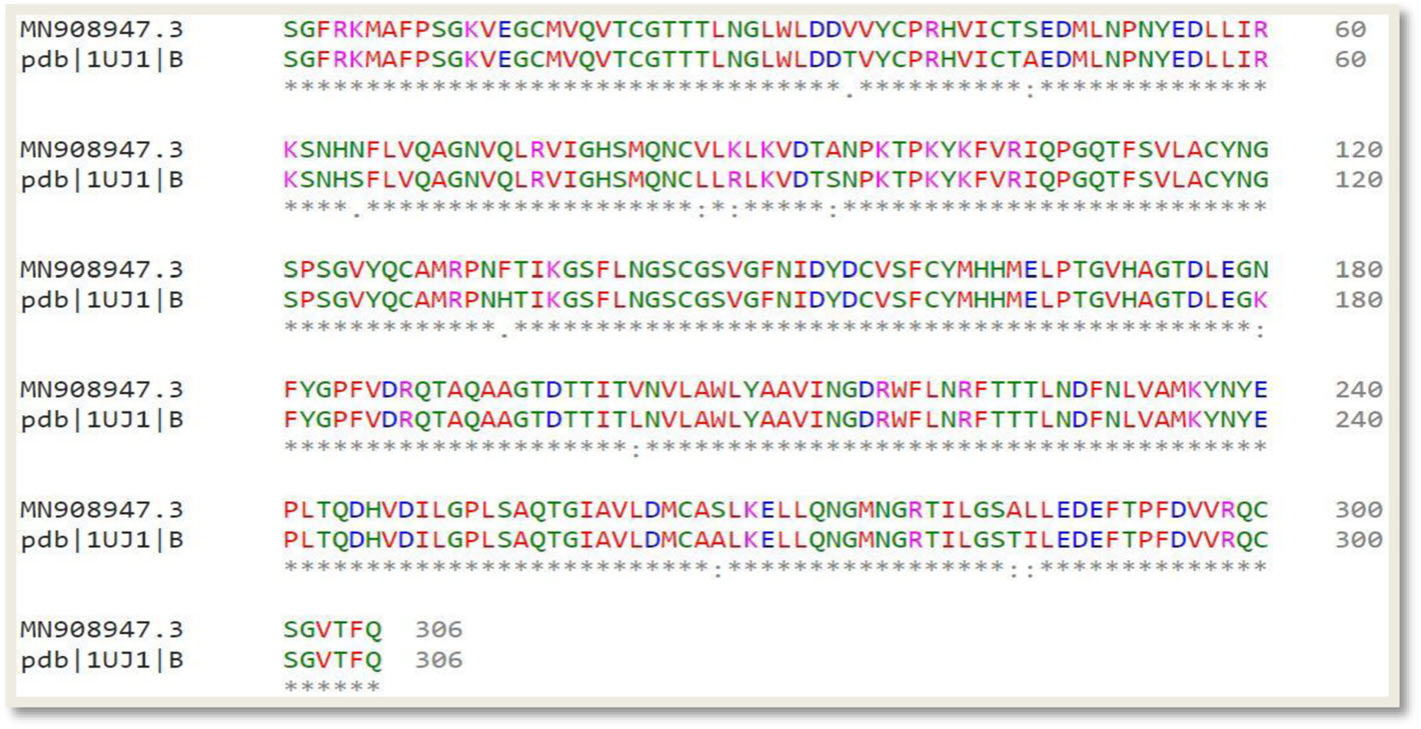
Sequence alignment between the amino acid sequence of the target protein and the SARS coronavirus main proteinase

As depicted in Fig. 3, few structural differences were noticed. The amino acid sequences making up these non-conserved regions were clearly revealed in Fig. 4. Notwithstanding, a 96% identity was observed between both sequences showing the conserved domains were predominant. Figure 4 represents the percentage amino acid sequence identity between the target and the template protein, where the positions with a single asterisk (*) depicts regions of full residue conservation while the segments with the colon (:) indicates regions of conservation between amino acid residues with similar properties. Positions with the period (.) show regions of conservation between amino acids with less similar properties.

**Fig. 5:**
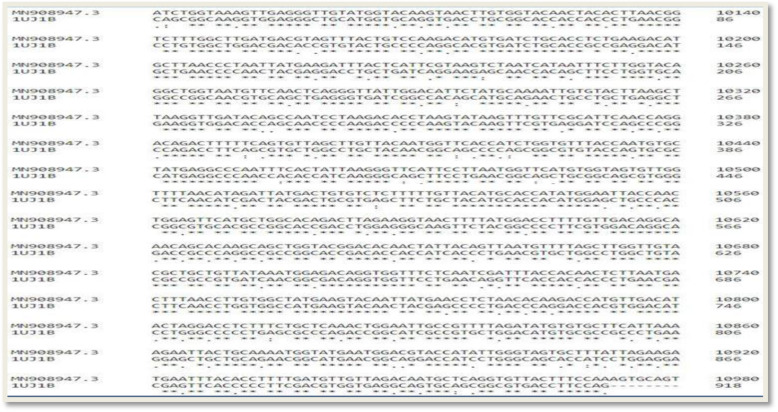
Sequence alignment between the nucleotide sequence of the back-translated SARS coronavirus main proteinase and the 10055 to 10972 nucleotide region of the 2019-nCoV complete genome

The amino acid sequence of the SARS coronavirus main proteinase was back-translated to generate the corresponding nucleotide sequence which was then aligned with the 2019-nCoV full genome. This was carried out for the purpose of mapping out the region of the 2019-nCoV full genome where the proteinase coding sequence is located. As depicted in Fig. 5, the target protein coding sequence is located between 10055 and 10972 nucleotides of the viral genome

### QMEAN

**Fig. 6:**
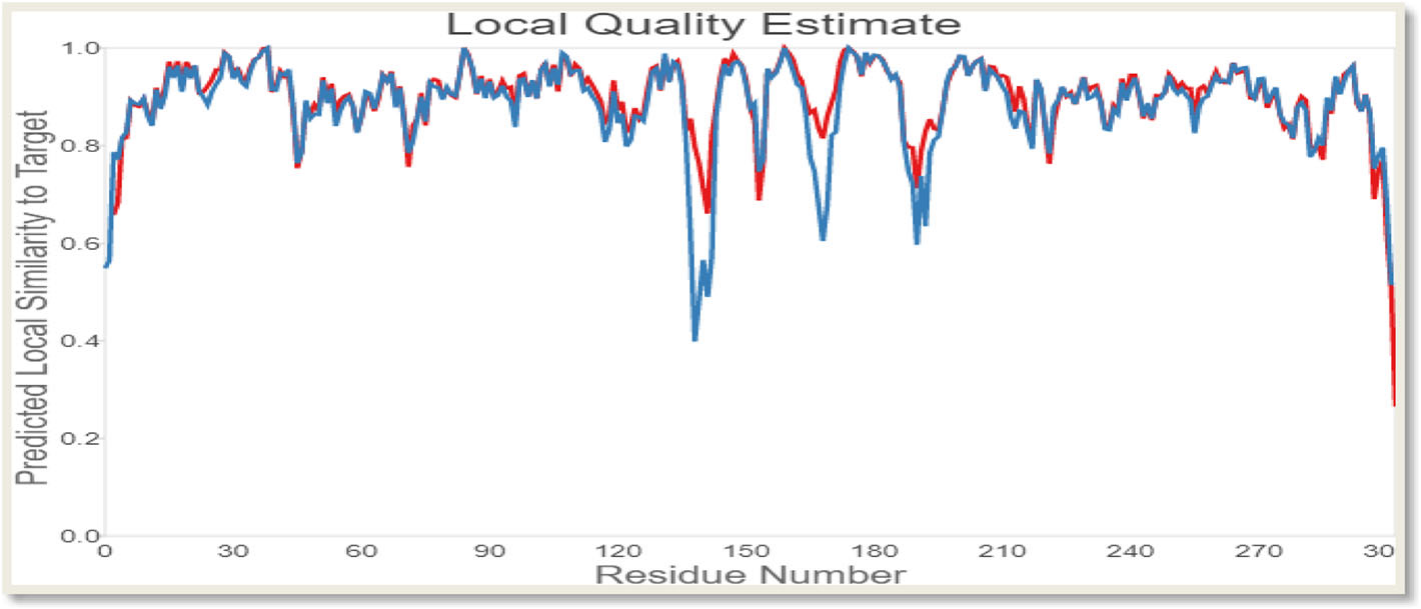
Local quality estimate graph showing the values of the predicted local similarity to target (*y*-axis) plotted against the protein residue number (*x*-axis). This quality estimation was carried out on both chains of the target protein with the red and blue lines representing chains A and B, respectively

The outcome of a QMEAN analysis is anchored on the composite scoring function which calculates several features regarding the structure of the target protein. The expression of the estimated absolute quality of the model is in terms of how well the score of the model is in agreement with the values expected of a set of resultant structures from high-resolution experiments. The global score values can either be from QMEAN4 or QMEAN6. QMEAN4 is a combination of four statistical potential terms represented in a linear form while QMEAN6 in addition to the functionality of QMEAN4 uses two agreement terms in the consistency evaluation of structural features with sequence-based predictions. Both QMEAN4 and QMEAN6 originally are in the range of 0 to 1 with 1 being the good score and are by default transformed into *Z*-scores (Table 1) for them to be related to what would have been expected from X-ray structures of high resolution. The local scores are also a combination of four linear potential statistical terms with the agreement terms of evaluation being on a per residue basis. The local scores are also estimated in the range of 0 to 1, where one is the good score (Fig. 6).

**Table 1:** Z-score for the individual components of QMEAN for the model protein

Components	Scores
QMEAN score	0.31
Interaction energy of C_β	− 0.35
Pairwise energy of all atoms	− 0.65
Solvation energy	− 0.77
Torsion angle energy	0.36

**Fig. 7:**
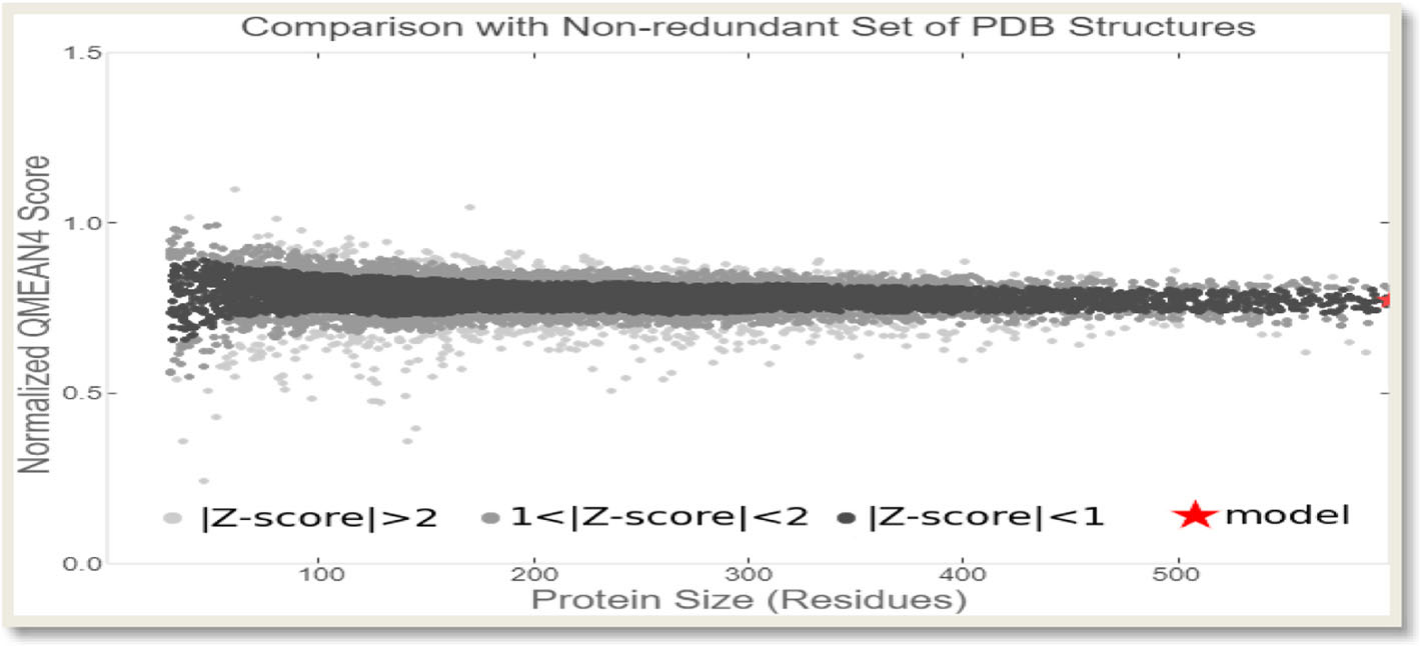
Graphical presentation of estimation of absolute quality of the target protein

When compared to the set of non-redundant protein structures, the QMEAN *Z*-score of the target protein as shown in Fig. 7 was 0. The models located in the dark zone are shown in the graph to have scores less than 1 while the scores of other regions outside the dark zone can either be 1 < the *Z*-score < 2 or *Z*-score > 2. Good models are often located in the dark zone.

### Ramachandran plot

**Fig. 8:**
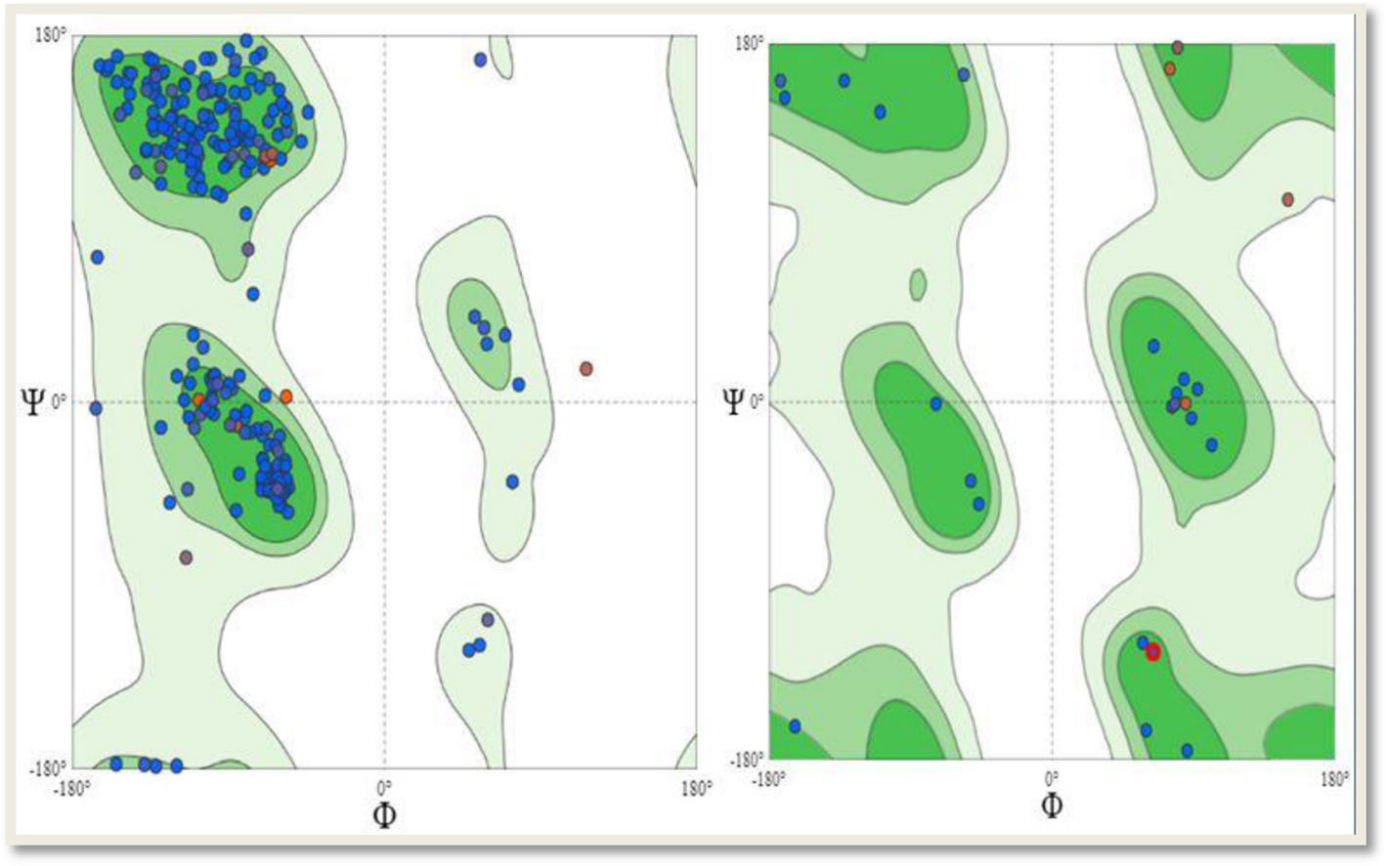
Depicted here are two Ramachandran plots. The plot on the left hand side shows the general torsion angles for all the residues in the target protein while the plot on the right hand side is specific for the glycine residues of the protein

The Ramachandran angles restriction in the target protein to specific values is shown in the Ramachandran plot displayed in Fig. 8. The plot displays the characteristic range of φ and ψ angles occupied by each type of secondary structure elements where the φ values are outlined on the horizontal axis while the ψ values are outlined on the vertical axis. The dots on the plot indicate the amino acid angles and counting is initiated from the left corner of the plot which ranges from − 180^°^ extending to + 180^°^ across both the horizontal and vertical axis. This allows for the clear distinction of the regions characterizing the secondary structure components. The low-energy regions on the plot are those segments with the highest dot density. These regions are also regarded as the allowed regions. Steric clash occurs at some values of φ and ψ because at these values, atoms are brought too close to each other and as such these values are regarded as forbidden. For experimental structures of high resolution, the forbidden regions are either almost empty or completely empty, and only a few amino acid residues have their torsion angles therein, although this rule accommodates some exemptions. Whenever such values are found, they result in some strains in the polypeptide chain and in cases of such, the stability of the structure will depend greatly on additional interactions but this conformation may be conserved in a protein family for its structural significance. Another α- and β-regions clustering principle exemption can be viewed on the right side plot of Fig. 8 where the distribution of torsion angles for glycine are the only displayed angles on the Ramachandran plot. Glycine has no side chain, and this gives room for flexibility in the polypeptide chain hence making accessible the forbidden rotation angles. Glycine for this reason is more concentrated in the regions making up the loop where sharp bends can occur in the polypeptide. For this reason, glycine is highly conserved in protein families as the presence of turns at specific positions is a characteristic feature of particular structural folds.

### Computation of physiochemical properties

**Fig. 9:**
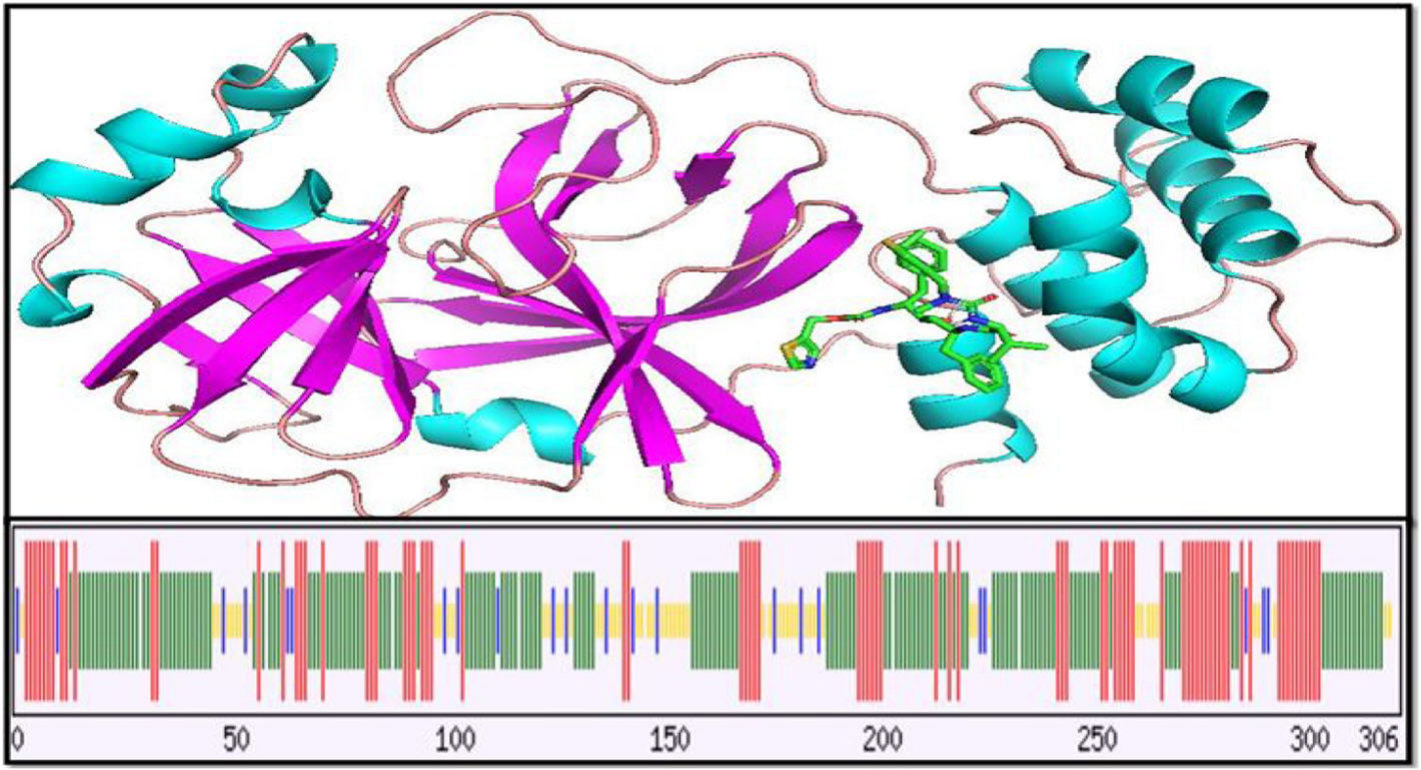
The target protein secondary structures with bound lopinavir. At the top is the secondary structure visualization on pymol with regions making up the alpha helix, beta sheets, and loops shown in light blue, purple, and brown, respectively. Below is the prediction by CFSSP where the red, green, yellow, and blue lines depict regions of the helices, sheets, turns, and coils (loops), respectively. The predicted secondary structure composition shows a high degree of alpha helix and beta sheets, respectively, occupying 45 and 47% of the total residues with the percentage loop occupancy at 8%

**Fig. 10:**
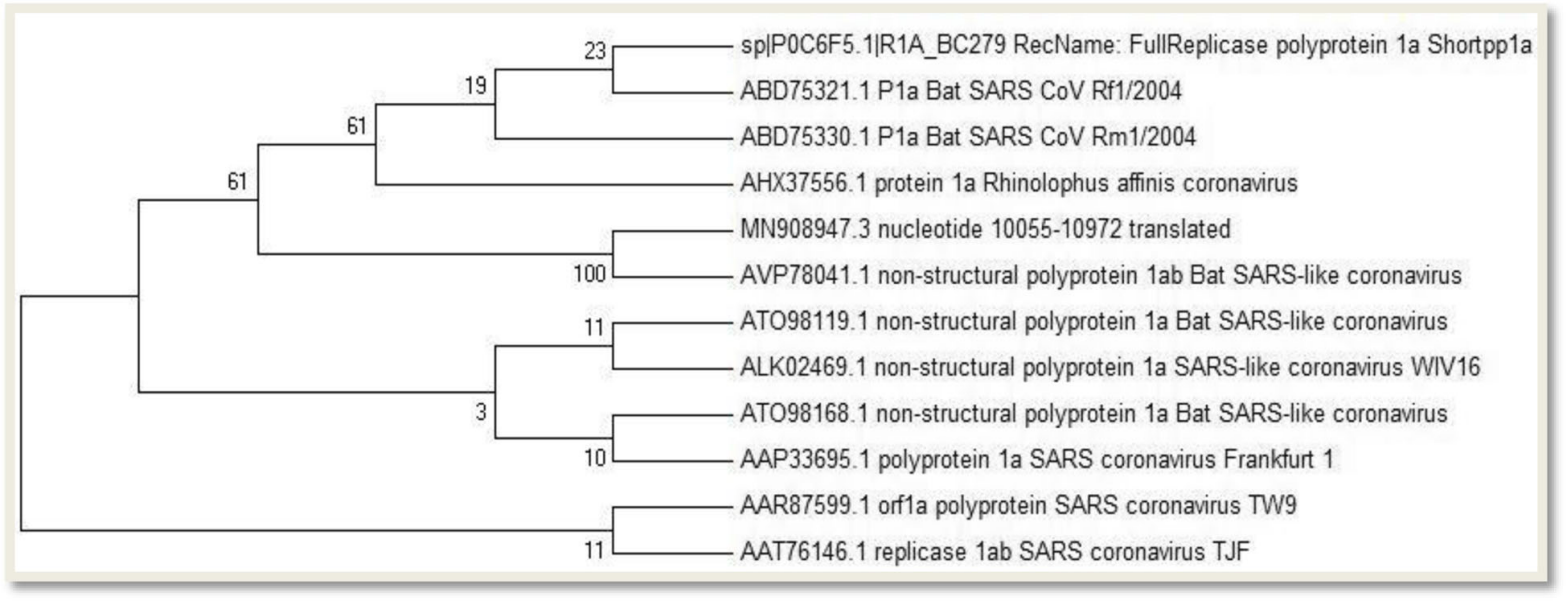
Bootstrap consensus phylogenetic tree

The comparative physiochemical parameter computation of the template and target proteins by ProtParam were deduced from the amino acid sequences of the individual proteins. No additional information was required about the proteins under consideration and each of the complete sequences was analyzed. The amino acid sequence of the target protein has not been deposited in the Swiss-Prot database. For this reason, inputting the standard single-letter amino acid sequence for both proteins into the text field was employed in computing the physiochemical properties as shown in Tables 3, 4 and Figs. 9, 10.

Figure 10 depicts the maximum likelihood phylogenetic tree constructed based on 12 different amino acid sequences and showing the evolutionary relationship between the 2019-nCoV modeled main proteinase and other strains of closely related proteins. Bootstrap values (expressed as percentage of 1000 replications) are shown at the branching points of the tree. With a bar of 0.005 substitutions per site, SARS coronavirus polyprotein and replicase (strain TW9 and TJF) appeared as the out group.

### Molecular docking

**Fig. 11:**
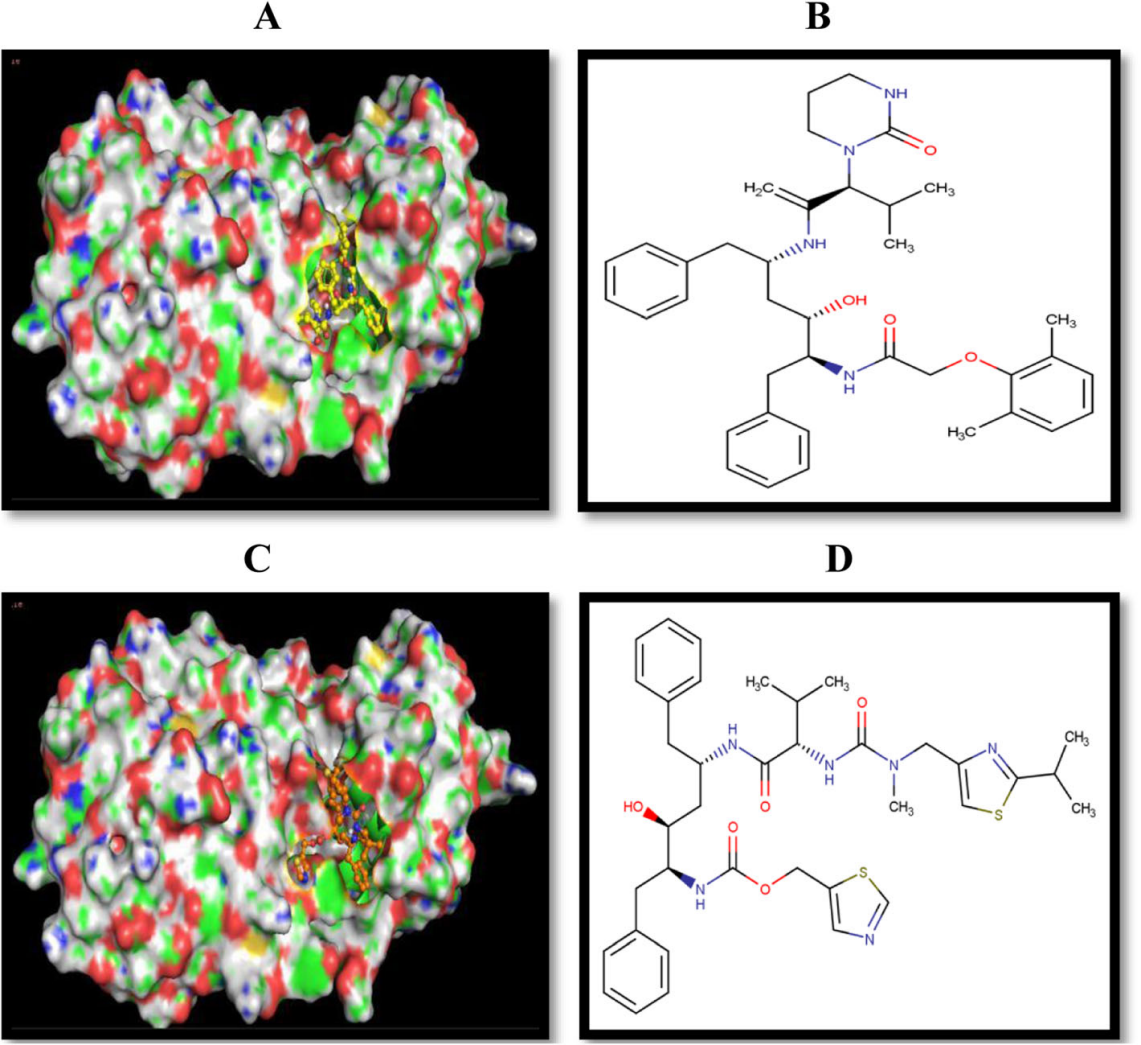
**a**, **c** The induced fit binding of lopinavir and ritonavir respectively to the target protein active site while **b** and **d** show their respective 2D structures

**Fig. 12:**
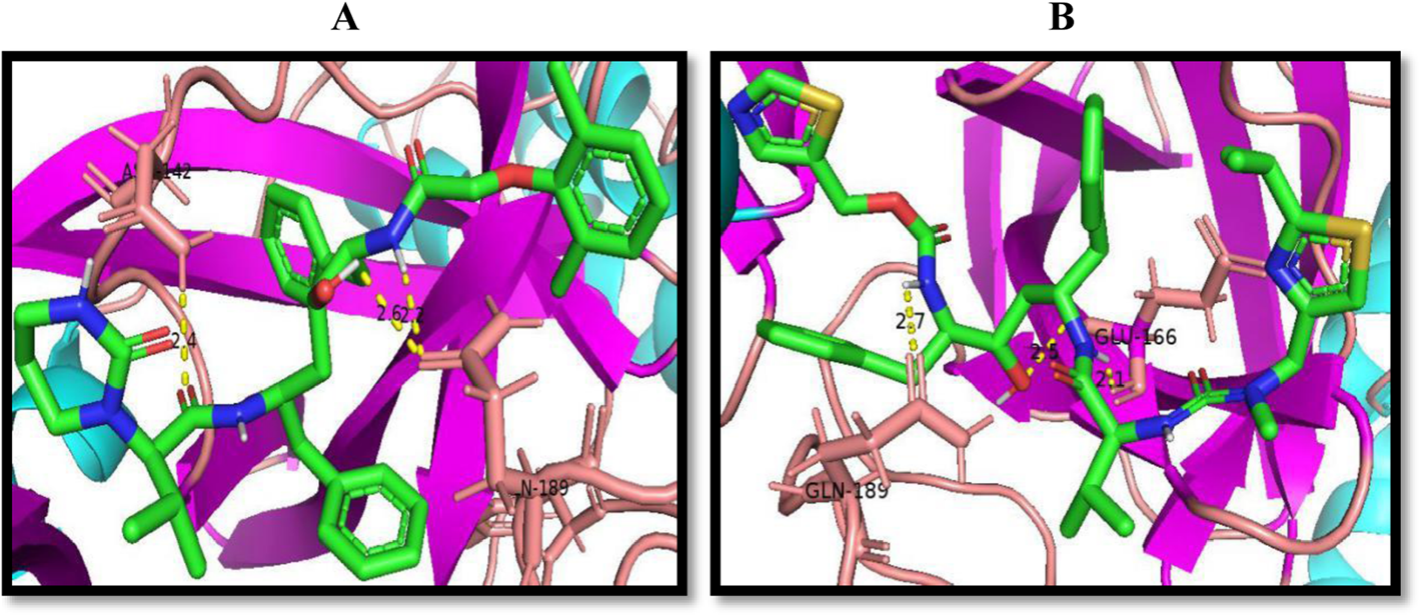
The polar interaction of lopinavir (**a**) and ritonavir (**b**) when bound to the active site of the downloaded 3D structure of the SARS-CoV main proteinase (6m2n). The compounds are colored predominantly in green (according to elements) for each column and bound to the active site residues displayed in a zoomed ribbon form and colored according to secondary structures (regions of helix, sheets, and coils colored in blue, purple, and brown, respectively)

The two HIV protease inhibitors (lopinavir and ritonavir) when targeted at the modeled 2019-nCoV catalytic site gave significant inhibition attributes; hence, the in silico study was planned through molecular docking analysis with AutoDock Vina. The binding orientation of the drugs to the protein active site as viewed in the pymol molecular visualizer (Fig. 11) showed an induced fit model binding conformation. The same compounds were targeted against the active site of the downloaded PDB 3D structure of the SARS-CoV main proteinase (PDB 6m2n) for comparison purposes Fig. 12.

**Fig. 13:**
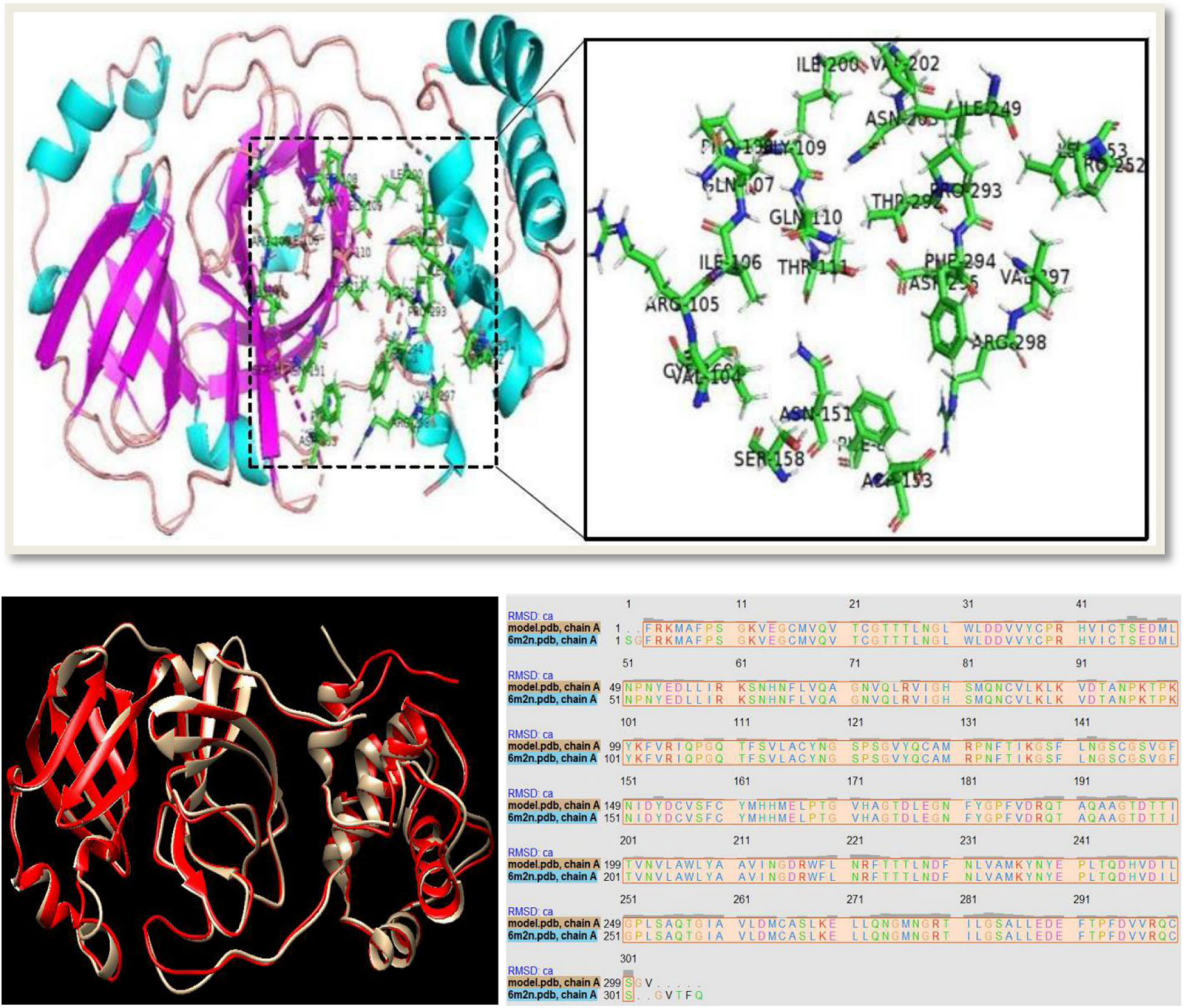
Amino acid residues forming the target protein active site

The active site residues as visualized in PyMol are shown in Fig. 13. The binding of lopinavir to the target protein which produced the best binding score was used as the predictive model. Residues at the distance of < 5 angstroms to the bound ligand were assumed to form the active site residues. A total of 27 amino acid residues were covered and these are PHE-8, VAL-104, ARG-105, ILE-106, GLN-107, PRO-108, GLY-109, GLN-110, THR-111, ASN-151, ASP-153, SER-158, CYS-160, ILE-200, VAL-202, ASN-203, HIS-246, PRO-252, ILE-249, PRO-252, LEU-253, THR-292, PRO-293, PHE-294, ASP-295, VAL-297, and ARG-298.

Fig. 13 The combined view of the 3D structural comparison between the modeled target protein and the downloaded PDB structure of the viral protein (left column) and their primary sequence alignment (right column). The target protein is colored in grey while its protein data bank equivalence is colored in red. The high structural similarity between the two proteins was validated through their sequence alignment which produced 99.34% sequence identity score.

## Discussion

Homology modeling which is a computational method for modeling the 3D structure of proteins and also regarded as comparative modeling, constructs atomic models based on known structures or structures that have been determined experimentally and likewise share more than 40% sequence homology. The backing principle for this is that there is likely to be an existing three-dimensional structure similarity between two proteins with high similarity in their amino acid sequence. With one of the proteins having an already determined 3D structure, the structure of the unknown one can be copied with a high degree of confidence. There is a higher degree of accuracy for alpha carbon positions in homology modeling than the side chains and regions containing the loop which is mostly inaccurate. As regards the template selection, homologous proteins with determined structures are searched through the Protein Data Bank (PDB) and templates must have alongside a minimum of 40% identity with the target sequence, the highest possible resolution with appropriate cofactors for selection consideration [29]. In this study, the target protein was modeled using the SARS coronavirus main proteinase as template. This selection was based on the high resolution and its identity with the target protein which is as high as 96%.

Qualitative Model Energy Analysis (QMEAN) is a composite scoring function that describes protein structures on the basis of major geometrical aspects. The scoring function of the QMEAN calculates the global quality of models on six structural descriptors linear combination basis, where four of the six descriptors are statistical potentials of mean force. The analysis of local geometry is carried out by potential of torsion angle over three consecutive amino acids. In predicting the structure of a protein structure, final models are often selected after the production of a considerable number of alternative models; hence, the prediction of the protein structure is anchored on a scoring function which identifies within a collection of alternative models the best structural model. Two distance-dependent interaction potentials are used to assess long-range interactions based on C_β atoms and all atoms, respectively. The burial status of amino acid residues describes the solvation potential of the model while the two terms that reflect the correlation between solvent accessibility, calculated and predicted secondary structure are not excluded [38]. The resultant target protein can be considered a good model as the *Z*-scores of interaction energy of C_β, pairwise energy of all atoms, solvation energy, and torsion angle energy are − 0.35, − 0.65, − 0.77, and 0.36, respectively, as shown in Table 1. The quality of a good model protein can be compared to high-resolution reference structures that are products of X-ray crystallography analysis through *Z*-score where 0 is the average *Z*-score value for a good model [26]. According to Benkert et al., QMEAN *Z*-score provides an estimate value of the degree of nativeness of the structural features that can be observed in a model, and this is an indication that the model is of a good quality in comparison to other experimental structures [26]. Our study shows the *Z*-score of the target is “0” as indicated in Fig. 6 and such a score is an indication of a relatively good model as it possesses the average *Z*-score for a perfect model.

Properties of the model that is predicted determines the MolProbity scores. Work initially done on all-atom contact analysis has shown that proteins possess exquisitely well-packed structures with favorable van der Waals interactions which overlap between atoms that do not form hydrogen bonds [39]. Unfavorable steric clashes are correlated strongly with the quality of data that are often poor where a near zero occurrence of such steric clashes occurs in the ordered regions of crystal structures with high resolution. Therefore, low values of clash scores are indications of a very good model which likewise has been proven by the clash score value exhibited by the target protein that was modeled for the purpose of this study (Table 2). In addition to the clash score, the protein conformation details are remarkably relaxed, such as staggered *χ* angles and even staggered methyl groups [40]. Applied forces to a given local motif in environments predominantly made up of folded protein interior can produce a locally strained conformation but significant strain are kept near functionally needed minimum by evolution and this is on the presumption that the stability of proteins is too marginal for high tolerance. In traditional validation measures updates, there has been a compilation of rigorously quality-filtered crystal structures through homology, resolution, and the overall score validation at file level, by B-factor and sometimes at residue level, by all-atom steric clashes. The resulting multi-dimensional distributions generated after an adequate smoothing are used in scoring the “protein-like” nature of each local conformation in relation to known structures either for backbone Ramachandran values or the side chain rotamers [41]. Rotamer outliers are equivalent to < 1% at high resolution while general-case Ramachandran outliers to a high-resolution equivalence of < 0.05%, and Ramachandran favored to 98%. In this regard, the definition of the MolProbity score (MPscore) was given as

**Table 2:** The individual parameters and scores as calculated by MolProbity

Parameters	Scores
MolProbity score	1.82
Clash score	2.06
Ramachandran favored	95.66%
Ramachandran outliers	0.83%
Rotamer outliers	5.21%

MPscore = 0.426 *ln(1+clashscore) + 0.33 *ln(1+max(0, rota_out|-1)) + 0.25 *ln(1+max(0, rama_iffy|-2)) + 0.5

Where the number of unfavorable all-atom steric overlaps ≥ 0.4 Å per 1000 atoms defines the clashscore [38]. The rota_out is the side chain conformation percentage termed as the rotamer outliers, from side chains that can undergo possible evaluation while rama_iffy is the backbone Ramachandran percentage conformations that allows beyond the favored region, from residues that can undergo possible evaluation. The derivatives of the coefficients are from a log-linear fit to crystallographic resolution on a set of PDB structures that has undergone filtration, so that the MPscore of a model is the resolution at which each of its scores will be the values expected thus, the lower MPscores are the indications of a better model. With a clash score of 2.06 and a 95.66% value for the Ramachandran favored region as compared to the Ramachandran outliers and rotamer outliers with individual values of 0.83% and 5.21% respectively, we arrived at a MolProbity score of 1.82 which is low enough to indicate the quality of a good model in our experimental protein.

The characteristic repetitive conformation attribute of amino acid residues is the basis for the repetitive nature of the secondary structures hence the repetitive scores of φ and ψ. The range of φ and ψ scores can be used in distinguishing the different secondary structural elements as the different φ and ψ values of each secondary structure elements map their respective regions on the Ramachandran plot. Peptides of the Ramachandran plot have the average values of their α-helices clustered about the range of φ = − 57^°^ and ψ = − 47^°^ while the average values of 130^°^ and ψ = + 140^°^ describes the Ramachandran plot clustering for twisted beta sheets [42]. The core region (green in Fig. 8) on the plot has the most favorable combinations for the φ and ψ values and has the highest number of dots. The figure also shows in the upper right quadrant, a small third core region. This is known as the allowed region and can be found in the areas of the core regions or might not be associated with the core region. It has lesser data points compared to the core regions. The other areas on the plot are regarded as disallowed. Since glycine has only one hydrogen atom as side chain, steric hindrance is not as likely to occur as φ and ψ are rotated through a series of values. The glycine residues having φ and ψ values of + 55^°^ and − 116^°^, respectively [43] do not exhibit steric hindrance and for that reason positioned in the disallowed region of the Ramachandran plot as shown in the right hand side plot in Fig. 8.

The extinction coefficient is an indication of the intensity of absorbed light by a protein at specific wavelength. The importance of estimating this coefficient is to monitor a protein undergoing purification in a spectrophotometer. Woody in his experiment [44] has shown the possibility of estimating a protein’s molar extension coefficient from knowledge of its amino acid composition which has been presented in Table 3.

**Table 3:** Amino acid composition table for both template and target protein

Amino acid residues in one letter codes																				
Proteins	A	R	N	D	C	Q	E	G	H	I	L	K	M	F	P	S	T	W	Y	V
Template	17	12	19	17	12	14	9	26	8	12	30	11	10	16	13	16	26	3	11	24
Target	17	11	21	17	12	14	9	26	7	11	29	11	10	17	13	16	24	3	11	27

The extinction coefficient of the proteins (both the template and the target proteins) was calculated using the equation:

E(Prot) = Numb(Tyr) × Ext(Tyr) + Numb(Trp) × Ext(Trp) + Numb(Cystine) × Ext(Cystine)The absorbance (optical density) was calculated using the following formula:

$$ \mathrm{Absorb}\left(\mathrm{Prot}\right)=\mathrm{E}\left(\mathrm{Prot}\right)/\mathrm{Molecular}\_\mathrm{weight} $$

For this equation to be valid, the following conditions must be met: pH 6.5, 6.0 M guanidium hydrochloride, 0.02 M phosphate buffer.

The N-terminal residue identity of a protein is an important factor in the determination of its stability in vivo and also plays a major role in the proteolytic degradation process mediated by ubiquitin [45]. β-galactosidase proteins with different N-terminal amino acids were designed through site-directed mutagenesis, and the designed β-galactosidase proteins have different half-lives in vivo which is striking, ranging from over a hundred hours to less than 2 min, but this is dependent on the nature of the amino terminus residue on the experimental model (yeast in vivo; mammalian reticulocytes in vitro, E. coli in vivo). The order of individual amino acid residues is thus in respect to the conferred half-lives when located at the protein’s amino terminus [46]. This is referred to as the “N-end rule” which was what the estimated half-life of both the template and target proteins were based on. The instability index provides an estimate of the protein’s stability in a test tube. Statistical analysis of 32 stable and 12 unstable proteins has shown [47] that there are specific dipeptides with significantly varied occurrence in the unstable proteins as compared with those in the stable proteins. The authors of this method have assigned a weight value of instability to each of the 400 different dipeptides (DIWV). The computation of a protein’s instability index is thus possible using these weight values, which is defined as:

$$ {\displaystyle \begin{array}{c}\mathrm{i}=\mathrm{L}\hbox{-} 1\\ {} II=\left(10/L\right)\times Sum\  DIWV\left(x\left[i\right]x\left[i+1\right]\right)\\ {}\mathrm{i}=1\end{array}} $$

Where L is the sequence length and DIWV(x[i]x[i + 1]) is the instability weight value for the dipeptide starting from position i.

A protein that exhibits an instability index value less than 40 can be predicted as a stable protein while an instability index value that exceeds the 40 threshold is an indication that the protein may be unstable. The comparative instability index values for the template and target proteins were 29.67 and 27.65 (Table 4), respectively, showing both are stable proteins. The relative volume occupied by aliphatic side chains (valine, alanine, leucine, and isoleucine) of a protein is known as its aliphatic index. It may be an indicator of a positive factor for an increment in globular proteins thermostability. The aliphatic index of the experimental proteins was calculated according to the following formula [48]:

$$ \mathrm{Aliphatic}\ \mathrm{index}=\mathrm{X}\left(\mathrm{Ala}\right)+\mathrm{a}\times \mathrm{X}\left(\mathrm{Val}\right)+\mathrm{b}\times \left[\mathrm{X}\left(\mathrm{Ile}\right)+\mathrm{X}\left(\mathrm{Leu}\right)\right] $$

**Table 4:** Calculated physiochemical properties by the ExPASy ProtParam server

Calculated parameters	Template protein	Target protein
Molecular weight	33845.72	33796.64
Theoretical *pI*	6.22	5.95
Amino acid composition (total)	306	306
Atomic composition	C_1499_H_2325_N_405_O_445_S_22_	C_1499_H_2318_N_402_O_445_S_22_
Extinction coefficient	33640	33640
Estimated half-life	30 h (mammalian reticulocytes, in vitro).	30 h (mammalian reticulocytes, in vitro).
	> 20 h (yeast, in vivo).	> 20 h (yeast, in vivo).
	> 10 h (Escherichia coli, in vivo).	>10 h (Escherichia coli, in vivo).
Instability index	29.67	27.65
Aliphatic index	81.83	82.12
GRAVY	− 0.049	− 0.019

Where X(Ala), X(Val), X(Ile), and X(Leu) are the mole percent (100 × mole fraction) of alanine, valine, isoleucine, and leucine. The coefficients “*a*” and “*b*” are the relative volume of the valine side chain (*a* = 2.9) and of Leu/Ile side chains (*b* = 3.9) to the alanine side chain. The calculated aliphatic index for the experimental protein shows that the thermostability of the target protein is slightly higher than the template.

The most common secondary structures are the alpha helices and beta sheets although the beta turns and omega loops also occur. Elements of the secondary structures spontaneously form as an intermediate before their folding into the corresponding three-dimensional tertiary structures [49]. The stability and how robust the α-helices are to mutations in natural proteins have been shown in previous studies. They have also been shown to be more designable than the beta sheets; thus, designing a functional all-α helix protein is likely to be easier than designing proteins with both α helix and strands, and this has recently been confirmed experimentally [50]. The template and target proteins both have a total of 306 amino acid residues (Table 4) with the composition of individual residues shown in Table 3. As shown in Fig. 9, the target protein which shares a structural homology with the template (Fig. 3 and the animation video) is predominantly occupied by residues forming alpha helix and beta sheets, with very low percentage of the residues forming loops. The stability of these two proteins is revealed in their physiochemical characteristics which can therefore be linked to the high percentage of residues forming alpha helix.

The ultimate goal of genome analysis is to understand the biology of organisms in both evolutionary and functional terms, and this involves the combination of different data from various sources [51]. For the purpose of this study, we compared our protein of interest to similar proteins in the NCBI database to predict the evolutionary relationships between homologous proteins represented in the genomes of each divergent species. This makes the amino acid sequence alignment the most suitable form of alignment for the phylogenetic tree construction. Organisms with common ancestors were positioned in the same monophyletic group in the tree, and the same node where the protein of interest (the 2019-nCoV main proteinase) is positioned also houses the non-structural polyprotein of the 1ab Bat SARS-like coronavirus. This shows that the two viral proteins share a common source with shorter divergence period. Bootstrapping allows evolutionary predictions on the level of confidence. One hundred is a very high level of confidence in the positioning of the node in the topology. The lower scores are more likely to happen by chance than it is in the real tree topology [52]. The bootstrap value of the above-mentioned viral proteins which is exactly 100 is a very high level of statistical support for their positioning at the nodes in the branched part of the tree. The length of the branches is a representation of genetic distance. It is also the measure of the time since the viral proteins diverged, which means, the greater the branch length, the likelihood that it took a longer period of time since divergence from the most closely related protein [53]. The TW9 and TJF strains of the SARS coronavirus orf1a polyprotein and replicase, respectively, are the most distantly related, based on their branch length and as such can be regarded as the out-group in the tree.

Structure-based drug discovery is the easiest molecular docking methodology as it screens variety of listed ligands (compounds) in chemical library by “docking” them against proteins of known structures which in this study is the modeled 3D structure of the 2019-nCoV main proteinase and showing the binding affinity details alongside the binding conformation of ligands in the enzyme active site [54]. Ligand docking can be specific, that is, focusing only on the predicted binding sites of the protein of interest or can be blind docking where the entire area of the protein is covered. Most docking tool applications focus on the predicted primary binding site of ligands; however, in some cases, the information about the target protein binding site is missing. Blind docking is known to be an unbiased molecular docking approach as it scans the protein structure in order to locate the ideal binding site of ligands [55]. The AutoDock-based blind docking approach was introduced in this study to search the entire surface of the target and template protein for binding sites while optimizing the conformation of peptides simultaneously. For this reason, it was necessary to set up our docking parameters to search the entire surface of the modeled main proteinase of the 2019-nCoV. This was achieved using the AutoGrid to create a very large grid map (center 77 Å × − 10 Å × 15 Å and size 30 Å × 60 Å × 35 Å) with the maximum number of points in each dimension in order to cover the whole protein. We observed a partial overlap in the docking pose of lopinavir to the active site of both template and target protein as compared to the conspicuous difference observed in the binding orientation of ritonavir to the protein active sites. These differential poses can be viewed distinctively in the attached animation video. A keen view of the binding orientation of the two drug candidates to the 2019-nCoV virus main proteinase active site (Fig. 11) is also consistent with the proposed induced fit binding model. In a comparative docking study, the same drug candidates (lopinavir and ritonavir) were docked against the active site of the PDB downloaded version of the viral main proteinase. The docking grid for this purpose was set with precision as the solved PDB structure of the virus included a co-crystalized ligand at the enzyme active site (center – 32 Å × − 65 Å × 42 Å and size 25 Å × 30 Å × 25 Å) and experimental ligands bind to this site with precision and variation in poses (Fig. 12). The binding energy results showed a difference of − 0.3 Kcal/mol upon the binding of lopinavir to the template and the PDB 3D structure of the enzyme (PDB 6m2n), and a difference of − 0.5 Kcal/mol between the PDB 3D structure of the enzyme and the target protein (Table 5 and 6). The same comparative study was repeated for the binding of ritonavir and a difference of − 0.1 and − 1.0 Kcal/mol was observed upon the binding of drug to the template and target proteins, respectively, in comparison with the binding to the downloaded 3D structure of the enzyme from the PDB. The observed consistency in the binding energy of the drug candidates can also serve as a reference to the validity and quality of the modeled protein, which has exhibited a high sequence and structural similarity with the downloaded 3D structure from the protein data bank (Fig. 13).

**Table 5:** Here, the docking results of lopinavir and ritonavir against the template and target protein are shown. The binding of ritonavir to the template protein produced the highest number of inter model hydrogen bonds while the binding of lopinavir to the target protein formed polar interaction with three residues at the active site as compared to the two formed by the other interactions

S/N	HIV protease inhibitors	Canonical SMILES	Proteins	Polar interactions	Inter model hydrogen bonds	Docking score (Kcal/mol)
1	Lopinavir	CC1=C(C(=CC=C1)C)OCC(=O)NC(CC2=CC=CC=C2)C(CC(CC3=CC=CC=C3)NC(=O)C(C(C)C)N4CCCNC4=O)O	Template	GLN-110, PHE-294	16	− 8.1
			Target	GLN-110, THR-292, PHE-294	19	− 8.3
2	Ritonavir	CC(C)C1=NC(=CS1)CN(C)C(=O)NC(C(C)C)C(=O)NC(CC2=CC=CC=C2)CC(C(CC3=CC=CC=C3)NC(=O)OCC4=CN=CS4)O	Template	GLN-110, SER-158	20	− 6.7
			Target	THR-292, PHE-294	19	− 7.8

**Table 6:** The amino acid residues involved in polar interaction, the number of inter-model hydrogen bonds and the docking score of lopinavir and ritonavir upon binding to the 3D PDB download of the SARS-CoV main proteinase (PDB 6m2n)

Compounds	Polar interactions	Inter-model hydrogen bonds	Docking score (Kcal/mol)
Lopinavir	142-ASN, 189-GLN	16	− 7.8
Ritonavir	166-GLU, 189-GLN	20	− 6.8

## Conclusion

In an effort to make available potent therapeutic agents against the fast rising 2019 novel coronavirus epidemic, we identified from the viral genome the coding region and modeled the main proteinase of the virus coupled with the evaluation of the efficacy of existing HIV protease inhibitors by targeting the protein active site using a blind docking approach. Our study has shown that lopinavir displays a broader spectrum inhibition against both the SARS coronavirus and 2019-nCoV main proteinase as compared to the inhibition profile of ritonavir. The modeled 3D structure of the enzyme has also provided interesting insights regarding the binding orientation of the experimental drugs and possible interactions at the protein active site. The conclusion from the study of Cao et al. as previously discussed however has shown that the administration of the lopinavir-ritonavir therapy might elicit additional health concerns as a result of the extreme adverse events exhibited by the experimental subjects for the purpose of their study. It was also observed that the drugs showed no increased benefit when compared with the standard supportive care. In view of this findings, we therefore suggest a drug modification approach aimed at avoiding the health concerns posed by the lopinavir-ritonavir combined therapy while retaining their proteinase inhibitory activity.

## Supplementary information

**Additional file 1.** Supplementary information to this article can be found online at https://www.rcsb.org/structure/6M2N

## Data Availability

Not applicable

## Abbreviations

PDB: Protein Data Bank
3CLpro: 3C-like protease
SARS-CoV: Severe Acute Respiratory Syndrome Coronavirus
2019-nCoV: 2019 novel coronavirus
ARD: Acute respiratory disease
RNA: Ribonucleic acid
hCoV: Human coronavirus
TGEV: Transmissible gastroenteritis virus
HIV: Human immunodeficiency virus
AIDS: Acquired immunodeficiency syndrome
NCBI: National Center for Biotechnology Information
INSDC: International Nucleotide Sequence Database Collaboration
SVM: Support vector machines
QSQE: Quaternary structure quality estimate
CFSSP: Chou and Fasman Secondary Structure Prediction
JTT: Jones-Taylor-Thornton
MEGA: Molecular Evolutionary and Genetics Analysis
SMILES: Simplified Molecular Input Line Entry System
RMSD: Root mean square deviation
QMEAN: Qualitative Model Energy Analysis
GRAVY: Grand average of hydropathy
